# Supplementary Low Far-Red Light Promotes Proliferation and Photosynthetic Capacity of Blueberry In Vitro Plantlets

**DOI:** 10.3390/ijms25020688

**Published:** 2024-01-05

**Authors:** Yuting Wang, Zhehao Jiang, Wenxiang Li, Xiaolong Yang, Chengming Li, Dunxu Cai, Yunxue Pan, Wei Su, Riyuan Chen

**Affiliations:** College of Horticulture, South China Agricultural University, Guangzhou 510642, China; wangyt@stu.scau.edu.cn (Y.W.); zhjiang@stu.scau.edu.cn (Z.J.); wxli@stu.scau.edu.cn (W.L.); yxl0214@scau.edu.cn (X.Y.); 20213137124@stu.scau.edu.cn (C.L.); 20213137099@stu.scau.edu.cn (D.C.); panyunxue@stu.scau.edu.cn (Y.P.)

**Keywords:** *Vaccinium*, far-red light, photosynthetic traits, proliferation, transcriptomics, WGCNA

## Abstract

Far-red light exerts an important regulatory influence on plant growth and development. However, the mechanisms underlying far-red light regulation of morphogenesis and photosynthetic characteristics in blueberry plantlets in vitro have remained elusive. Here, physiological and transcriptomic analyses were conducted on blueberry plantlets in vitro supplemented with far-red light. The results indicated that supplementation with low far-red light, such as 6 μmol m^−2^ s^−1^ and 14 μmol m^−2^ s^−1^ far-red (6FR and 14FR) light treatments, significantly increased proliferation-related indicators, including shoot length, shoot number, gibberellin A3, and trans-zeatin riboside content. It was found that 6FR and 14 FR significantly reduced chlorophyll content in blueberry plantlets but enhanced electron transport rates. Weighted correlation network analysis (WGCNA) showed the enrichment of iron ion-related genes in modules associated with photosynthesis. Genes such as *NAC*, *ABCG11*, *GASA1*, and *Erf74* were significantly enriched within the proliferation-related module. Taken together, we conclude that low far-red light can promote the proliferative capacity of blueberry plantlets in vitro by affecting hormone pathways and the formation of secondary cell walls, concurrently regulating chlorophyll content and iron ion homeostasis to affect photosynthetic capacity.

## 1. Introduction

Light is one of the most critical environmental factors influencing plant growth and development [1]. It generates energy through photosynthesis and serves as a signaling regulator of photomorphogenesis in plants [2,3]. In recent years, far-red light (701–750 nm) has been recognized as being crucial for photosynthesis [4,5,6]. The addition of far-red light to white light enhances canopy photosynthesis [4]. The solar spectrum is more conducive to exciting photosystem II (PSII), whereas far-red light is more prone to overstimulation of photosystem I (PSI) [7,8]. A balanced combination of different wavelengths is important for optimizing photosynthetic efficiency by regulating the activity of PSII and PSI. Plant phytochromes are the primary photoreceptors for red and far-red light and play a crucial role in regulating plant growth and development [3,9]. When the red-to-far-red light ratio is low, signals perceived by phytochrome B (phyB) induce shade avoidance syndrome (SAS), characterized by stem elongation, reduced branching, and altered leaf development [10].

Chlorophyll influences light absorption, whereas carotenoids engage in photoprotection to reduce photodamage and play a crucial role in plant photosynthetic capacity [11]. Chlorophyll fluorescence is used to monitor photosynthesis in plants [12]. Studies conducted on soybean, tomato, and lettuce have shown that treatments with a low red-to-far-red ratio decrease chlorophyll content in plants but increase the net photosynthetic rate [13,14,15]. Abundant far-red light accelerates rapid cyclic electron transfer around PSI, thereby safeguarding PSI [16,17]. The addition of far-red light to warm white light significantly enhances the quantum yield of photosystem II in lettuce, reduces non-photochemical quenching (NPQ), and diminishes thermal dissipation [18].

Far-red light has exhibited multifaceted effects on plant morphology. Supplementing far-red light (3 W·m^−2^ and 6 W·m^−2^) in white light conditions (200 μmol m^−2^ s^−1^) significantly increased plant height, internode length, stem diameter, and biomass in Chinese kale (*Brassica alboglabra* Bailey) [19]. A low red/far-red light ratio induced elongation of hypocotyl cells in *Brassica rapa* [20]. In wheat (*Triticum aestivum* L.), supplementation of natural radiation with far-red light (15 μmol m^−2^ s^−1^) suppressed tiller number [21]. In red–blue light mixed conditions (200 μmol m^−2^ s^−1^), supplementing far-red light (50 μmol m^−2^ s^−1^) significantly increased the total biomass and leaf area of lettuce (*Lactuca sativa* L. cv. ‘Tiberius’) [15]. Total leaf area is dependent on the leaf area divided by leaf mass (SLA) and biomass allocation, with a higher tendency to allocate biomass to stems under high far-red light conditions [22,23].

A low red-to-far-red light ratio can modulate shoot branching and stem elongation in plants by regulating hormonal signals [24,25,26]. In *Pinus tabuliformis* seedlings, far-red light promoted the expression of flavin monooxygenase (*YUC*), leading to an increase in auxin content and stem elongation [27,28]. Far-red light influences plant morphology through the auxin-mediated brassinosteroid (BR) signaling pathway [29]. Within the gibberellin pathway, far-red light upregulates the expression of GA biosynthetic genes, specifically kaurenoic acid oxidase genes. This results in elevated GA levels that affect plant cell division and elongation, thereby modulating plant morphology [26,30]. In response to far-red light, most regulatory pathways or signals converge on cell growth mechanisms, influencing plant morphology [30]. Phytochrome-interacting factors (PIFs) have emerged as key genes that respond to far-red light, with PIFs in *Arabidopsis* directly targeting *XTH15* and affecting cell growth [31]. Xyloglucan endotrans glucosylase/hydrolase (XTH) encodes cell wall-modifying enzymes that are critical for processes involving cellulose, lignin, and hemicellulose during cell growth [32]. Genes such as *XTH17*, *XTH19*, and *XTR7* play crucial roles in far-red light-induced morphological changes in plants [33,34].

Blueberry (*Vaccinium* spp.) belongs to the Ericaceae family and is an economically important crop [35]. However, blueberries exhibit low propagation efficiency through cuttings. In vitro propagation provides a rapid and efficient means to generate large numbers of plants [36,37]. Tissue culture relies on artificial light sources, and the artificial light environment is one of the key factors in tissue culture [38]. Adjusting the light quality combination can result in different plant morphologies, which have strong implications for the optimization of in vitro seedling reproduction [39]. Morphogenesis and photosynthesis in blueberries are closely related to light quality. Several studies have reported on the effects of light quality on the morphology and physiology of blueberries. A red-to-blue light ratio of 2:1 increased stem length, photosynthetic pigment content, fresh weight, and peroxidase activity in in vitro seedlings of blueberries (*Vaccinium* spp.) [40]. Under light quality treatments for southern highbush blueberries (*Vaccinium corymbosum* L.), blue light treatment increased chlorophyll and anthocyanin content [41]. Blue light treatment induces the expression of *LDOX*, *OMT*, and *UFGT*, enhancing the anthocyanin content in blueberry leaves [42]. However, to date, most studies on the effects of light quality on the growth and development of blueberries have focused on red and blue light spectra. There has been relatively limited research integrating morphological and physiological–biochemical changes with transcriptome profiles to examine the impact of far-red LED on in vitro cultured blueberry plants. Here, based on the above analysis, we speculated that additional supplementary far-red light had an important effect on the proliferation and growth of blueberry tissue culture seedlings.

This study revealed the impact of supplementing different intensities of far-red (FR) light on the proliferation and photosynthetic capacity of blueberry in vitro plantlets. Additionally, the study utilized weighted correlation network analysis (WGCNA) to identify key candidate modules and genes regulated by far-red light in photosynthetic characteristics and proliferation. The findings of this study lay the foundation for further investigating the molecular mechanisms of far-red light regulation in plant photosynthesis and proliferation. Furthermore, the results provide insights into optimizing the light environment for enhancing the efficiency of genetic transformation and propagation in blueberries.

## 2. Results

### 2.1. Effects of Supplementing FR Light on Morphology 

**Figure 1:**
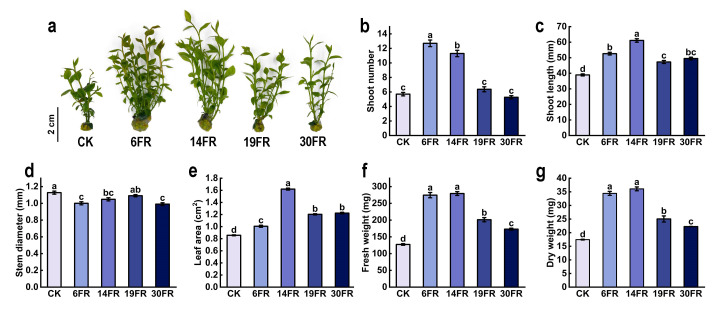
Morphological data of blueberry in vitro seedlings. (**a**) Plant morphology, (**b**) shoot number, (**c**) shoot length, (**d**) stem diameter, (**e**) leaf area, (**f**) fresh weight per seedling, and (**g**) dry weight per seedling. Data are presented as the mean ± standard deviation (*n* = 3). Adopting the Waller–Duncan’s test for significant differences (*p* ≤ 0.05), distinct letters represent significant differences. CK, 6FR, 14FR, 19FR, and 30FR represent far-red light supplementation levels of 0, 6, 14, 19, and 30 µmol m^−2^ s^−1^, respectively.

The effects of supplementing different intensities of far-red light on the morphology of blueberry seedlings are shown in Figure 1a. Compared with the control (CK), the shoot number significantly increased by 122.8% and 98.2% in the 6FR and 14FR treatments, respectively (Figure 1b). Among all the treatments, plants from the 6FR treatment had the highest number of shoots (Figure 1b). Shoot length in the treatments with FR light of different intensities was significantly greater than that in the CK treatment (Figure 1c). In the 14FR treatment, shoot length was the highest, showing a 56.9% increase compared to CK (Figure 1c). The stem diameter of plants subjected to FR light treatments showed a slight reduction compared to that of the CK group. However, the difference was relatively minor. (Figure 1d). Leaf area was the largest under the 14FR treatment, with a significant increase of 89.2% compared to CK (Figure 1e). There was no significant difference in biomass between the 6FR and 14FR treatments, and both were the highest (Figure 1f,g). Fresh weight significantly increased by 115.7% and 119.4% in the 6FR and 14FR treatments, respectively, compared with CK (Figure 1f). Dry weight also significantly increased in the 6FR and 14FR treatments by 97.4% and 106.9%, respectively, compared with CK (Figure 1g). Shoot number, shoot length, leaf area, and biomass showed an initial increase, followed by a decrease with increasing intensity of FR light (Figure 1b–e,g).

### 2.2. Effects of Supplementing FR Light on Endogenous Hormone Levels

**Figure 2:**
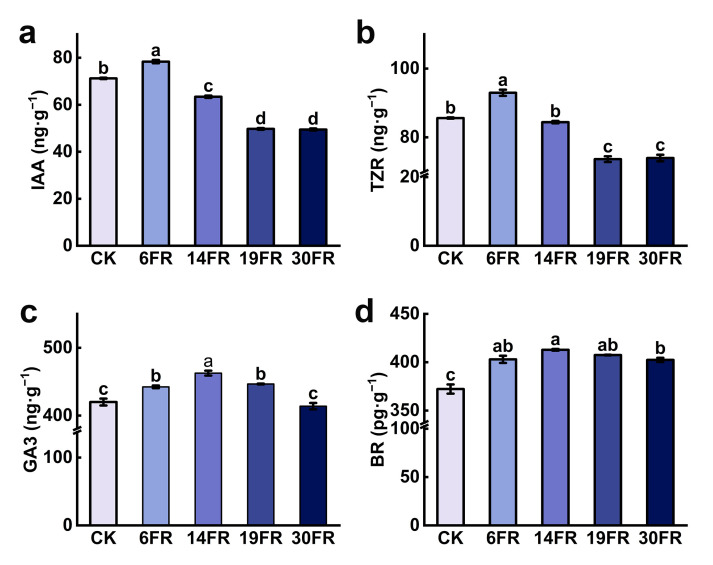
Endogenous hormone levels of blueberry in vitro plantlets. (**a**) IAA, indole-3-acetic acid; (**b**) TZR, trans-zeatin riboside; (**c**) GA3, gibberellin A3; (**d**) BR, brassinosteroids. Data are presented as the mean ± standard deviation (*n* = 3). Adopting the Waller–Duncan’s test for significant differences (*p* ≤ 0.05), distinct letters represent significant differences.

In vitro plantlets treated with 6FR exhibited the highest levels of IAA and trans-zeatin riboside (TZR). These were significantly increased by 10.0% and 8.6%, respectively, compared to those in CK (Figure 2a,b). The levels of IAA and TZR in plants treated with 14FR, 19FR, and 30FR were lower than those in the CK (Figure 2a,b). The BR content in in vitro blueberry plantlets exposed to FR light was significantly higher than that in the CK plantlets (Figure 2d). However, the differences among treatments were relatively small. In the 14FR treatment, in vitro plantlets exhibited the highest levels of BR and GA3, increasing by 10.9% and 10.2%, respectively, compared to CK (Figure 2c,d). With an increase in FR light intensity, the levels of IAA, TZ, RBR, and GA3 showed a trend of initially increasing, followed by a decrease (Figure 2).

### 2.3. Effects of Supplementing FR Light on Photosynthetic Traits

**Figure 3:**
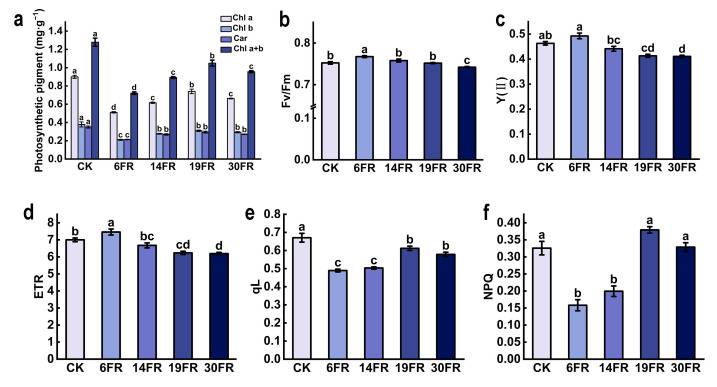
Photosynthetic characteristics of leaves of blueberry in vitro plantlets. (**a**) Photosynthetic pigment content; (**b**) Fv/Fm, maximal quantum yield of PSII; (**c**) Y(II), actual PSII quantum yield; (**d**) ETR, electron transport rates; (**e**) qL, fraction of open PSII centers based on a lake model; (**f**) NPQ, non-photochemical quenching; Data are presented as mean ± standard deviation. Data are presented as the mean ± standard deviation (*n* = 3). Adopting the Waller–Duncan’s test for significant differences (*p* ≤ 0.05), distinct letters represent significant differences.

The addition of FR light at different intensities significantly influenced the photosynthetic characteristics of blueberry plantlets in vitro. FR light supplementation resulted in a reduction in chlorophyll and carotenoid content (Figure 3a). The plants treated with 6FR exhibited the lowest levels of chlorophyll and carotenoids (Figure 3a). The values of Fv/Fm, Y(II), and ETR in the 6FR treatment were the highest. They showed a significant enhancement compared to CK, with increases of 2%, 6.3%, and 6.5%, respectively (Figure 3b–d). The values of qL and NPQ in the 6FR treatment were the lowest, exhibiting significant decreases of 27.0%, 51.4%, and 31.3%, respectively, compared to the control (Figure 3e,f). With increasing FR light intensity, the levels of chlorophyll, carotenoids, qL, and NPQ initially decreased and then increased (Figure 3a,e,f). As the FR light intensity increased, Fv/Fm, Y(II), and ETR showed an initial increase, followed by a decrease (Figure 3b–d).

### 2.4. Transcriptome Sequencing 

**Figure 4:**
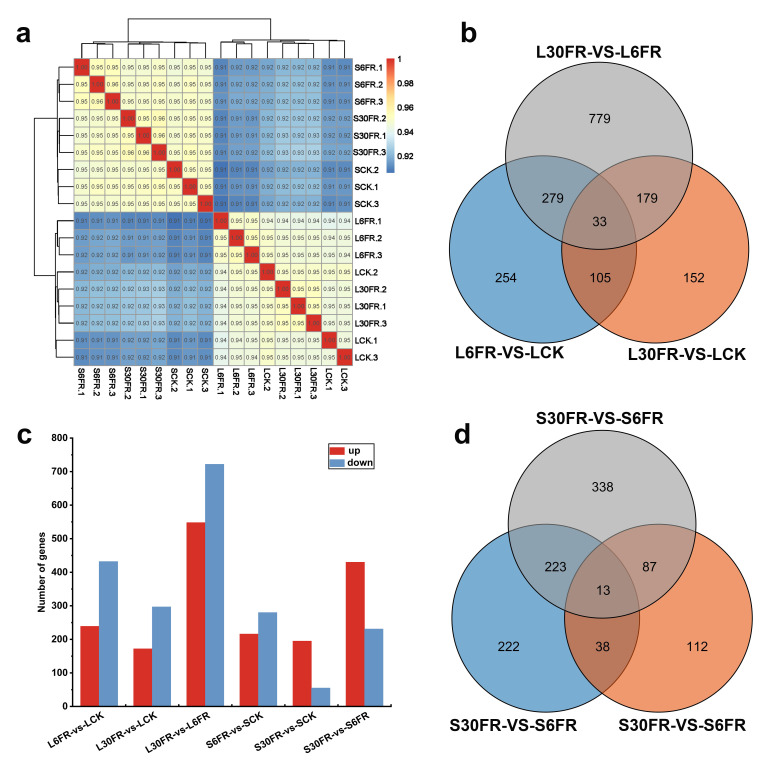
Global analysis of the blueberry leaf and stem transcriptomes. (**a**) Pearson correlation coefficients, (**b**) Venn diagram of DEGs in leaves, (**c**) number of upregulated and downregulated DEGs in leaves and stems, and (**d**) Venn diagram of DEGs in stems.

To elucidate the mechanisms by which FR light regulates the photosynthetic characteristics and morphogenesis of blueberry in vitro, samples were collected from the stems and leaves of blueberry plantlets subjected to the CK, 6FR, and 30FR treatments. Eighteen samples were subjected to transcriptome sequencing, yielding a cumulative total of 877.4 million clean reads. Each sample contained at least 36.9 million clean reads, with a Q30 base quality score of 91.8% or higher (Table S1). The Pearson correlation coefficient between the biological replicates for each treatment among the 18 samples exceeded 0.95. These results indicate the high quality of the RNA-seq data, rendering them suitable for downstream analyses (Figure 4a).

To identify differentially expressed genes (DEGs) among the samples under different treatment conditions, a comparative analysis was conducted. In total, 1781 non-redundant DEGs were identified in the differential comparison of leaf tissues. Comparison between the 30FR and 6FR light treatments resulted in the highest number of DEGs, including 722 downregulated and 548 upregulated DEGs (Figure 4b,c). A total of 1033 non-redundant DEGs were identified in the differential comparison of stem tissues. Similarly, the comparison between the 30FR and 6FR treatments showed the highest number of DEGs, including 231 downregulated and 430 upregulated DEGs (Figure 4c,d).

### 2.5. Co-Expression Network Analysis and Gene Ontology (GO) Analysis Shows the Impacts of FR Light on Proliferation 

**Figure 5:**
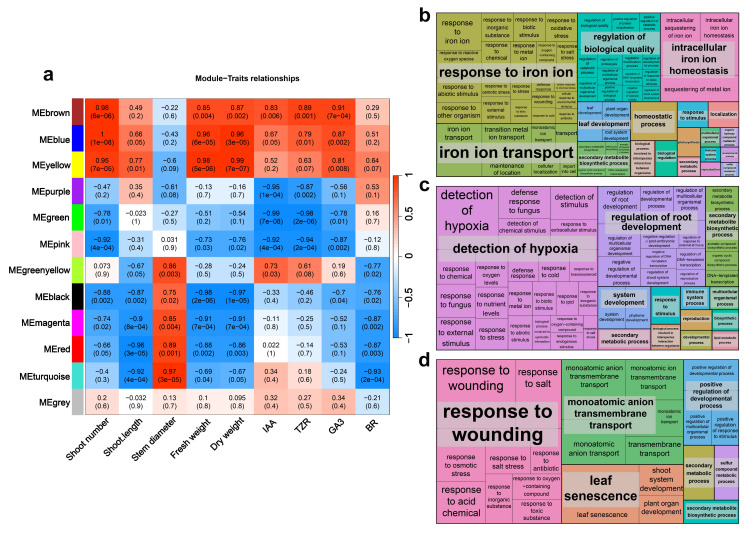
WGCNA analysis and GO enrichment analysis of the blueberry stem transcriptome. (**a**) Association between WGCNA modules and proliferation and hormone-related traits in the stem. The values in each square indicate the correlation coefficient between the module and the trait. (**b**) GO enrichment analysis results for the blue module. (**c**) GO enrichment analysis results for the yellow module. (**d**) GO enrichment analysis results for the brown module. The figures were generated using REVIGO, and each rectangle represents a cluster representative.

To identify key gene sets that influence different traits of in vitro blueberry plantlets under various FR light treatment conditions, we performed WGCNA on the identified DEGs in the stems (Figure S1). In the WGCNA of stems, the MEbrown, MEblue, and MEyellow modules exhibited strong positive correlations with traits such as shoot number, shoot length, stem diameter, fresh weight, and hormone content. Meanwhile, stem diameter showed an opposite association pattern with these indicators (Figure 5a). We conducted a GO enrichment analysis of the genes in the MEbrown, MEblue, and MEyellow modules (Figure 5b–d). Among these, 138 genes in the MEblue module were significantly enriched in biological processes, with particular attention paid to shoot system, plant organ, system, and anatomical structure development because of their relevance to stem development (Figure 5b, Table S2). To further explore the effects of FR light on stem development, we manually annotated the genes enriched in these four GO terms (Table S3). The results showed the inclusion of a considerable number of genes that are associated with auxin and cytokinin transport and cellulose synthesis. In GO enrichment analysis of MEyellow, 93 biological process terms were collectively enriched (Figure 5c, Table S4). These biological processes included GO terms related to stem development and plant hormones such as plant organ development, response to brassinosteroids, anatomical structure development, shoot system development, regulation of shoot system development, and response to hormones (Tables S4 and S5). In contrast, the GO enrichment analysis of MEbrown identified only 28 enriched GO terms, with a significant proportion of members associated with the *NAC* gene family (Tables S6 and S7). The *NAC* family plays important roles in the synthesis of cellulose, hemicellulose, and lignin.

### 2.6. Co-Expression Network Analysis and GO Analysis Shows the Impacts of FR Light on Photosynthetic Traits

**Figure 6:**
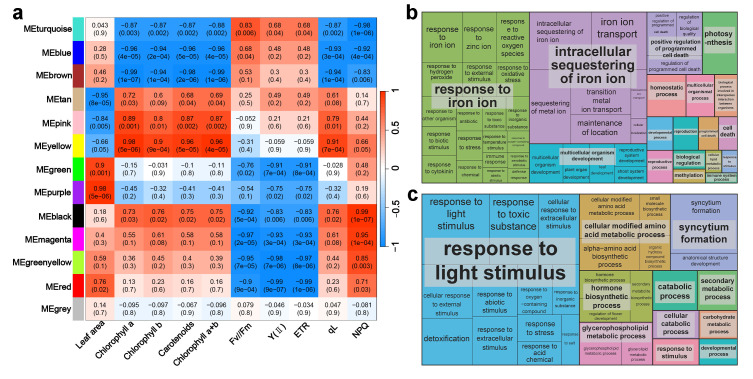
WGCNA analysis and GO enrichment analysis of the blueberry leaf transcriptome. (**a**) Association between WGCNA modules and proliferation and hormone-related traits in the stem. The values in each square indicate the correlation coefficient between the module and the trait. (**b**) GO enrichment analysis results for the magenta module. (**c**) GO enrichment analysis results for the yellow module. The figures were generated using REVIGO, and each rectangle represents a cluster representative.

We clustered DEGs in leaves through analysis of network topology and soft thresholding, and then correlated the clustering modules with phenotypic data (Figure S2). The 1781 non-redundant DEGs obtained from leaf tissue comparisons were subdivided into 13 modules (Figure 6a). Further correlation analysis between these modules and physiological indicators of leaves showed the strongest positive correlation between chlorophyll content and QL in the MEyellow module. Meanwhile, strong negative correlations were observed in the blue and brown modules (Figure 6a). The photosynthetic indicators of leaves (FV/FM, Y(II), and ETR) showed strong negative correlations with the magenta, green–yellow, and red modules (Figure 6a). NPQ, a chlorophyll fluorescence indicator, exhibited strong positive correlations with the black and magenta modules (Figure 6a). To further investigate the impact of FR light on photosynthesis in blueberry plantlets, we selected the MEmagenta and MEyellow modules from leaf WGCNA for GO enrichment analysis (Figure 6b,c). The MEmagenta module was enriched in 88 biological processes with many GO terms related to ion transport or storage, such as the intracellular sequestration of iron ions, sequestration of iron ions, iron ion transport, sequestration of metal ions, and obsolete iron ion homeostasis (Figure 6b, Table S8). Gene annotation results for these GO terms showed that most genes were FER genes (Table S9). In the yellow module, 46 biological processes were enriched, and terms such as response to light stimuli, radiation, and red or FR light indicated an association between the yellow module and leaf photosynthesis (Figure 6c, Table S10). Further annotation results for some terms also highlighted their involvement in photosynthesis and the FR light response, including genes such as *PRR5*, *GSTU17*, and *FSD2* (Table S11).

### 2.7. Analysis of Key Gene Expression Levels in Leaves and RT-qPCR Validation

**Figure 7:**
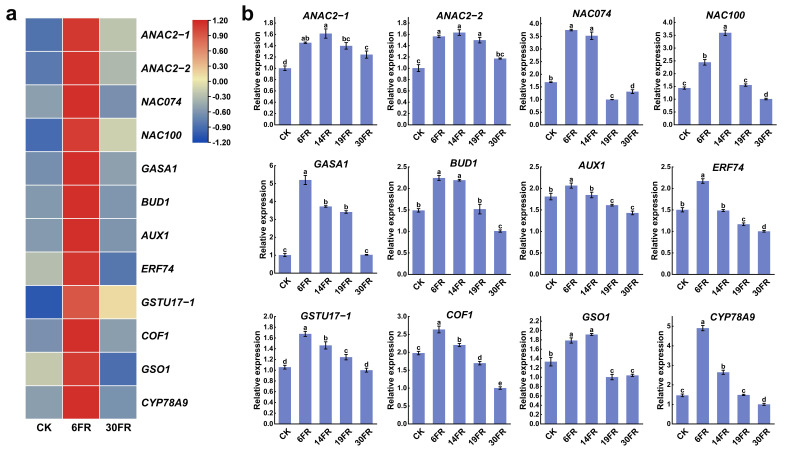
Expression analysis of stem proliferation candidate genes under far-red light treatment. (**a**) Heatmap of candidate genes based on RNA sequencing results. (**b**) RT−qPCR analysis of candidate genes associated with stem proliferation. Data are presented as the mean ± standard deviation (*n* = 3). Adopting the Waller–Duncan’s test for significant differences (*p* ≤ 0.05), distinct letters represent significant differences.

We conducted transcriptomic analysis of the selected DEGs identified in the stem GO analysis. Four members of the *NAC* family associated with the formation of secondary cell walls were significantly upregulated under treatment with a low level of far-red light (6FR), displaying an initial increase followed by a decrease with increasing far-red light intensity (Figure 7a). The expression patterns of these genes were consistent with the results obtained from the RT-qPCR analysis (Figure 7b). Genes related to gibberellin (GA), such as *GASA*, and genes associated with auxin, including *BUD1*, *AUX1*, *ERF74*, *GSTU17-1*, and *COF1*, were significantly upregulated by 6FR (Figure 7a). In the RT-qPCR analysis, upon additional supplementation with FR light, all these genes exhibited a downregulation trend with increasing FR light intensity (Figure 7b). These findings suggest that FR light regulates the proliferation of blueberry plantlets in vitro by influencing the formation of secondary cell walls and hormonal pathways.

### 2.8. Analysis of Key Gene Expression Levels in Stems and RT-qPCR Validation

**Figure 8:**
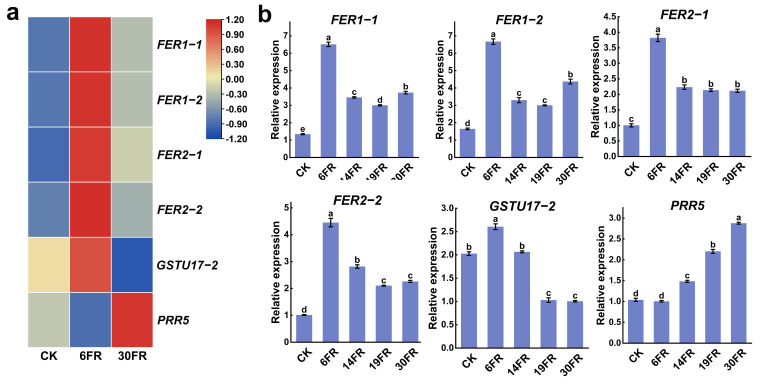
Expression analysis of candidate genes associated with photosynthesis in response to far-red light treatment. (**a**) Transcriptomic analysis of candidate genes associated with photosynthesis in leaves based on RNA sequencing results. (**b**) RT−qPCR analysis of candidate genes associated with photosynthesis in leaves. Data are presented as the mean ± standard deviation (*n* = 3). Adopting the Waller−Duncan’s test for significant differences (*p* ≤ 0.05), distinct letters represent significant differences.

We performed transcriptomic analysis of the selected DEG identified in the leaf GO analysis. Four FER genes associated with iron homeostasis were significantly upregulated after additional supplementation with FR light but showed a decreasing trend with an increase in FR light intensity (Figure 8b). *GSTU17−2* was significantly upregulated in the 6FR treatment, whereas it was downregulated in the 19FR and 30FR treatments (Figure 8b). In contrast, PRR5 showed continuous upregulation with increasing FR light. The RT-qPCR results for these genes were consistent with the transcriptome data (Figure 8a). These findings suggest that FER genes play a crucial role in the photosynthetic impact of FR light on blueberry plantlets in vitro.

## 3. Discussion

### 3.1. Impacts of Supplemental FR Light on the Photosynthetic Characteristics and Proliferation of Blueberry Tissue Cultured Seedlings

Supplemental FR light can reduce the chlorophyll content and enhance the net photosynthetic rate [14,15]. In rice mutants with reduced chlorophyll content, the net photosynthetic rate and electron transport are elevated, primarily because of the negligible impact of chlorophyll content limitation on photosynthesis under light-saturated conditions, compared to the increased photosynthetic capacity [43]. In this study, plants subjected to FR light treatment had lower chlorophyll content than control plants. The 6FR-treated plants had higher values for Fv/Fm, Y(II), and ETR than the control. Meanwhile, qL and NPQ were lower than those of the control. Supplementation with a certain amount of FR light reduces chlorophyll content while stimulating photosynthetic capacity, thereby enhancing the plant’s absorption of light energy.

Supplementation with far-red light can promote stem elongation, while reducing branching [44]. Similarly, in in vitro blueberry plantlets treated with 6FR and 14FR, significant promotion of stem elongation was observed. GA and BR play positive roles in far-red light-induced morphogenesis [45]. In rice, the SAS response is suppressed by PAC (a GA inhibitor) and PCZ (a BR inhibitor). Rice mutants with enhanced GA accumulation and BR sensitivity exhibit an increased SAS responses [46,47,48]. In the present study, plant height exhibited a trend similar to that of the concentrations of GA3 and BR, suggesting that FR light may influence stem elongation via the GA and BR pathways. Under far-red light, tomatoes inhibited CK and BR synthesis, thereby suppressing lateral branch development [49]. The 6FR treatment promoted the branching of blueberry plantlets in vitro. The concentrations of IAA and TZR showed a trend similar to that of the number of tillers, indicating that IAA and TZR may play crucial roles in FR light-mediated tillering.

### 3.2. Transcriptome Analysis Reveals Potential Regulatory Mechanisms of FR Light on Blueberry Proliferation

WGCNA has become a crucial tool in plant transcriptome analysis. It has been widely used for clustering differentially expressed genes into co-expression modules and linking these modules to the traits of interest [50,51,52]. In the present study, the proliferation indicators of blueberry tissue cultures were primarily associated with three modules, that is, yellow, blue, and brown. In the GO enrichment analysis of the brown module, four GO terms, shoot system development, plant organ development, system development, and anatomical structure development, were significantly enriched. Most blueberry genes enriched in these GO terms were homologues of the *Arabidopsis NAC* family members. *NAC* significantly contributes to the regulation of secondary cell wall (SCW) cell types [53,54]. SCWs is composed primarily of cellulose, lignin, and hemicellulose. The *Arabidopsis* NAC family members, *SND1* and *NST1,* positively regulate the formation of fiber secondary walls. *SND1* can also promote the expression of the *MYB46* gene, inducing significant upregulation of secondary wall-related genes, thereby promoting the deposition of cellulose, hemicellulose, lignin, and SCW in cells [55,56,57]. *Arabidopsis SND1/2/3/4/5* redundantly regulates SCW development [58]. All the reported NAC family members positively regulate SCW formation [54]. The expression levels of blueberry genes homologous to the *Arabidopsis* NAC were positively correlated with plant morphology. This suggests that blueberry NAC family members may be widely involved in the response of blueberry in vitro plantlets to FR light and may play a crucial role in their proliferation. 

In the blue module of the stem, *GASA1* (VaccDscaff303-augustus-gene-0.44) was significantly enriched. Its expression level was significantly increased under 6FR treatment. *Arabidopsis AtGASA6* was shown to integrate the GA, ABA, and glucose signaling pathways to regulate seed germination and hypocotyl elongation [59]. Potato *SN1* belongs to the *GASA* gene family, and silenced plants exhibit reduced leaf area and stunted growth due to altered cell division, cell wall composition, and primary metabolism [60]. The *GASA* family member *GhGEG* in *Gerbera hybrida* inhibits petal and cell elongation during petal development [61,62]. This illustrates the importance of GASA in plant cell and stem development, implying that VaccDscaff303-augustus-gene-0.44 may play a crucial role in stem elongation of blueberry in vitro seedlings induced by FR light. Some auxin-related genes homologous to *Arabidopsis* are also enriched in the blue module of the stem, such as *BUD1* (VaccDscaff14-processed-gene-313.4) and *AUX1* (VaccDscaff5-augustus-gene-212.22) [63,64,65]. The biosynthesis and transport of auxins play a critical role in shade avoidance responses [66,67]. The *PIN-FORMED 3* mutant in *Arabidopsis*, a regulator of auxin efflux, did not exhibit a shade avoidance response under low red to FR light ratios [68]. VaccDscaff14-processed-gene-313.4 and VaccDscaff5-augustus-gene-212.22 showed significantly increased expression under the 6FR treatment, indicating their crucial roles in far-red light-induced stem elongation.

In the yellow module, the homologous gene of *Arabidopsis ERF74*, VaccDscaff17-augustus-gene-368.23, exhibits interesting functions. Elevated expression of *Arabidopsis ERF74* reduces chlorophyll synthesis and promotes stem elongation [69]. Under low levels of far-red light (6FR) treatment, the expression of VaccDscaff17-augustus-gene-368.23, was significantly increased. The chlorophyll content in in vitro blueberry seedlings decreased, and the stem was elongated. This suggests that VaccDscaff17-augustus-gene-368.23 may play a crucial role in the response to FR light.

### 3.3. Transcriptome Analysis Reveals Potential Rregulatory Mechanisms of FR Light on the Photosynthetic Capacity of Blueberries

Iron is crucial for the structure and function of the photosynthetic electron transfer chain [70,71]. Severe iron deficiency impairs the normal functioning of Photosystem I (PSI), and iron homeostasis is important for plant growth, development, and photosynthesis [71,72]. Ferritin, a class of iron storage proteins, plays a vital role in maintaining iron homeostasis [73]. *Arabidopsis ferritin* mutant plants exhibit slow leaf growth, reduced carbon fixation, decreased biomass, and altered iron transport in the floral stalks [74]. Tomato *FER* mutants exhibit severe chlorosis under iron-deficient conditions and eventually die after the emergence of two to three small leaves [75]. The magenta module in the leaves was enriched with numerous biological processes related to ions or iron ions. Most of the enriched genes were homologous to *Arabidopsis FER*. These genes were induced to exhibit high expression levels under the 6FR treatment, which was also associated with improved photosynthetic traits. This suggests that FR light influences photosynthetic processes in micropropagated blueberry seedlings by modulating the expression of *FER* genes.

In the yellow module, which exhibited the highest correlation with chlorophyll content, particular attention was paid to genes homologous to *Arabidopsis GSTU17* (VaccDscaff3-processed-gene-167.17, VaccDscaff2-augustus-gene-270.35). *Arabidopsis GSTU17* responds to phytochrome A (phyA), thereby regulating FR light-mediated hypocotyl elongation and anthocyanin accumulation [76]. *Arabidopsis GSTU20* interacts with *FIN219* in the FR light signaling pathway, and the gain and partial loss of *GSTU20* function result in a decrease in the sensitivity of hypocotyls under continuous FR light [77,78]. Some *PRR* genes (VaccDscaff6-augustus-gene-247.26) were enriched in the yellow module. *PRR* genes participate in plant responses to FR light [79]. In *Arabidopsis*, *PRRs* interact directly with members of the *PIF* gene family, thereby inhibiting *PIF’s* ability of PIFs to regulate downstream genes [80]. In blueberry plantlets treated with 6FR, the homologue of *Arabidopsis PRR5*, VaccDscaff6-augustus-gene-247.26, was significantly downregulated. This is consistent with the response pattern of *PRR* to FR light in *Arabidopsis*. This suggests that the VaccDscaff6-augustus-gene-247.26 may also play a crucial role in the response of blueberry plantlets to FR light.

## 4. Materials and Methods

### 4.1. Plant Materials, Growth Conditions, and Light Experimental Design

The experiment was conducted in the culture room of the Baihua Garden at South China Agricultural University. In vitro plantlets of Southern Highbush Blueberry (*Vaccinium corymbosum* L.) ‘ZY09’ were used as the experimental material. In vitro blueberry plantlets were cultured in glass bottles containing 50 mL of woody plant medium (WPM), with a total volume of 300 mL per bottle. The culture medium comprised 20 g/L sucrose, 6 g/L agar, and 1 mg·L^−1^ Zeatin (ZT). Subculturing was performed every 60 d to ensure an adequate supply of materials for subsequent experiments. The experimental materials used in this study were in vitro seedlings that had undergone three rounds of subculturing. Stem segments of approximately 2 cm were excised from in vitro plantlets and placed in glass bottles containing WPM, with five plants per bottle. Each treatment contained 30 bottles with 150 plants. The cultivation room was maintained at a temperature of 25 ± 2 °C and a humidity level of 50–76%. The total light intensity for each treatment was set at 50 ± 2 μmol m^−2^ s^−1^, with a daily photoperiod of 16 h.

Light radiation was provided by an LED panel consisting of white light (460 ± 10 nm) and far-red (FR) light (730 ± 10 nm) LEDs (Guangzhou IGrowLite Agriculture Technology Co., Ltd., Guangzhou, China). Using 50 μmol m^−2^ s^−1^ white light as a control, treatments 6FR, 14FR, 19FR, and 30FR involved the addition of 6, 14, 19, and 30 μmol m^−2^ s^−1^ far-red light, respectively, on top of the control conditions. The total light intensities for the 6FR, 14FR, 19FR, and 30FR treatments were 51.1, 51.7, 52.3, 52.8 μmol m^−2^ s^−1^, respectively. After 40 d of cultivation, the blueberry tissue culture plantlets were harvested for subsequent parameter measurements. 

### 4.2. Chlorophyll Content and Chlorophyll Fluorescence Measurements

Photosynthetic pigments (chlorophyll a, chlorophyll b, and carotenoids) content was measured and calculated following methods described by Lichtenthaler [81]. Approximately 0.1 g of fresh leaves were weighed and added to a 10 mL test tube containing 2 mL anhydrous ethanol and 2 mL acetone. After 24 h in the dark, the samples of extraction solution were measured at 645 nm, 663 nm, and 440 nm using a UV-Vis spectrophotometer (Shimadzu UV-16A, Shimadzu, Corporation, Kyoto, Japan). Three biological replicates were performed for each treatment with 15 randomly selected blueberry plantlets. After 25 min of dark adaptation of the blueberry plantlets, chlorophyll fluorescence parameters were measured on the fifth leaf from the top using an Imaging-PAM chlorophyll fluorometer (WALZ, Nuremberg, Germany). The measured parameters included Fv/Fm (maximal quantum yield of PSII), Y(II) (actual PSII quantum yield), ETR (electron transport rates), qL (fraction of open PSII centers based on a lake model), NPQ (non-photochemical quenching), and qN (non-photochemical quenching coefficient).

### 4.3. Endogenous IAA, BR, GA3, and TZR Determination

We randomly selected fresh stem samples, ground them into powder in liquid nitrogen, weighed 100 mg, and then added them to 1 mL of phosphate-buffered saline (pH = 7.2–7.4). This was followed by an extraction at 4 °C in a refrigerator for 24 h. The next day, centrifugation at 1200× *g* was applied for 5 min, and the supernatant was collected. The levels of indole-3-acetic acid, brassinosteroids, gibberellin A3, and trans-zeatin riboside were determined following the instructions provided in the ELISA kit (Jiangsu Meibiao Biotechnology Co., Ltd., Yancheng, China).

### 4.4. RNA Extraction, cDNA Library Construction, and RNA-seq Analysis

For blueberry seedlings from each treatment, callus tissues were removed, and the stems and leaves were separated in liquid nitrogen using forceps. Subsequently, RNA was extracted separately from the leaves and stems. Total RNA was extracted using a DP441 RNAprep Pure Kit (TIANGEN, Beijing, China). We constructed cDNA libraries and performed RNA-seq separately for stems and leaves under different treatments. The experiments were conducted with the assistance of Guangzhou Genedenovo Biotechnology Co., Ltd. (Guangzhou, China; www.genedenovo.com). Raw sequencing data were evaluated for quality using FastQC v0.11.9 software (Bioinformatics Group at the Babraham Institute, Cambridge, UK) and low-quality data were filtered with Fastp version 0.23.4 using default parameters [82]. Clean reads were then mapped to the blueberry genome using hisat2 version 2.2.1 [83,84]. Subsequently, featureCount version 2.0.4 was used to obtain count values for each gene [85]. The transcripts per million (TPM) values were calculated using R version 4.2.3. The criteria for differential gene selection were set as |log2FoldChange| ≥ 1 and *p*-value ≤ 0.01.

### 4.5. WGCNA and GO Enrichment Analysis

Differential gene co-expression network analysis was performed using the R package weighted correlation network analysis (WGCNA) [86]. For leaf and stem tissues, 1033 and 1781 differential genes, respectively, were subjected to WGCNA. The soft-thresholding values were set to 25 and 18 with a minModuleSize of 30. These differential genes were clustered into 12 and 13 modules, respectively. The segmented modules were further correlated with the measured phenotypic data and visualized as a heat map depicting the correlations between the modules and traits. The phenotype data used by WGCNA are shown in Table S12.

For gene ontology (GO) annotation of the blueberry protein sequences, eggNOG-mapper v2 was used [87]. The GO enrichment program in the TBtools version 2.02 was used for GO enrichment analysis of genes within different modules identified by WGCNA [88]. The results of GO enrichment analysis were visualized using the REVIGO online platform [89].

### 4.6. Real-Time Quantitative PCR (RT-qPCR)

RT-qPCR was performed using SYBR Green Pro Taq HS (Accurate Biology, Changshang, China). RT-qPCR primers were designed using TBtools. The relative expression levels were calculated using the e 2^−∆∆Ct^ method. The RT-qPCR procedure was followed according to a previously established protocol [90]. *GAPDH* was used as the internal reference gene.

### 4.7. Statistical Analysis

Normal distribution tests and one-way analysis of variance (ANOVA) were performed using SPSS 20.0. Data are presented as the mean ± standard deviation (*n* = 3). The Waller–Duncan test was used to assess the significance of the mean differences among different treatments (*p* ≤ 0.05). Bar charts were generated using Origin 12.0.

## Data Availability

The data that support the findings of this study have been deposited into CNGB Sequence Archive (CNSA) of China National GeneBank DataBase (CNGBdb) with accession number CNP0005107.

## Supplementary Materials

The following supporting information can be downloaded at: https://www.mdpi.com/article/10.3390/ijms25020688/s1.
