# Supplementary material for: Supplementary Low Far-Red Light Promotes Proliferation and Photosynthetic Capacity of Blueberry In Vitro Plantlets
Source: Int J Mol Sci. 2024 Jan 5;25(2):688. doi: 10.3390/ijms25020688 (PMC10815622; doi:10.3390/ijms25020688)
Supplement: Supplementary file 1 [file ijms-25-00688-s001.zip › Supplementary Materials/Figure S2.pdf]

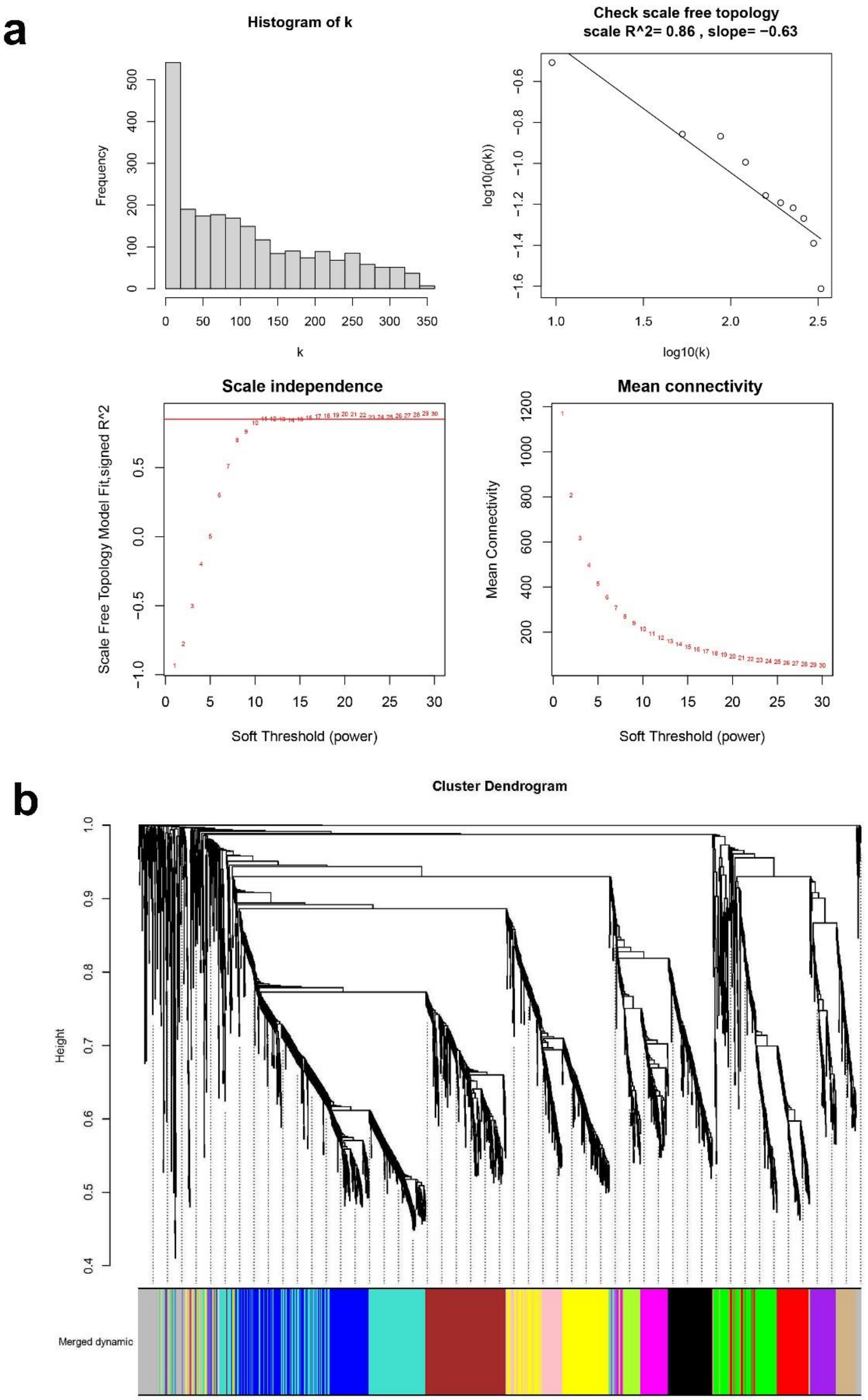

Figure S1. (a) Histogram of k, scale free topology scale, soft threshold and mean connectivity (b) Leaf differential genes clustered based on a dissimilarity measure (1-TOM)
